# Application of the Composite Fibers Based on Chitosan and Chitin Nanofibrils in Cosmetology

**DOI:** 10.3390/jfb13040198

**Published:** 2022-10-20

**Authors:** Vera V. Kodolova-Chukhontseva, Elena N. Dresvyanina, Yulia A. Nashchekina, Irina P. Dobrovol’skaya, Sergei G. Bystrov, Elena M. Ivan’kova, Vladimir E. Yudin, Pierfrancesco Morganti

**Affiliations:** 1 Institute of Biomedical Systems and Biotechnology, Peter the Great St. Petersburg Polytechnic University, Polytechnicheskaya Street 29, 195251 Saint Petersburg, Russia; 2 Institute of Textile and Fashion, Saint Petersburg State University of Industrial Technologies and Design, Bolshaya Morskaya Street 18, 191186 Saint Petersburg, Russia; 3 Institute of Cytology, Russian Academy of Sciences, Tikhoretsky Ave., 4, 194064 Saint Petersburg, Russia; 4 Udmurt Federal Research Center UB RAS, Tatiana Baramzina Street 34, 426067 Izhevsk, Russia; 5 Institute of Macromolecular Compounds, Bolshoy pr. 31, 199004 Saint Petersburg, Russia; 6 R&D Unit, Academy History of Healthcare Art, 00193 Rome, Italy

**Keywords:** serum, chitosan, chitin nanofibrils, filaments, wet spinning

## Abstract

Chitosan and composite fibers containing chitin nanofibrils have been developed for use in cosmetology. The tensile strength of the chitosan multifilaments is 160.6 ± 19.0 MPa, and of the composite multifilaments containing chitin, nanofibrils are 198.0 ± 18.4 MPa. Chitin nanofibrils introduced into the chitosan solution contribute to the creation of a new spatial arrangement of chitosan chains and their denser packing. The studies carried out by optical, scanning electron, and atomic force microscopy has shown that the serum, consisting of a mixture of lactic acid and sodium lactate, contains extended oriented structures—“liquid filaments”. It has been also shown that a mixture of serum and composite fibers based on chitosan and chitin nanofibrils has mucoadhesive, film-forming properties. The introduction of composite fibers containing chitin nanofibrils into the serum promotes the reinforcing effect of liquid filaments, the lifting effect of the film. The obtained composition can be used in cosmetology as a skin care product.

## 1. Introduction

Skin health is an important aspect of human well-being because the skin serves as the main barrier preventing microbial invasion [1]. Skin health is influenced by a few damaging factors [2]. Because of this, there is an increasing demand for biologically active products that can help preserve skin health and youth. One of the goals of cosmetic use is the saturation of protective tissues with minerals, vitamins, collagen, and other active compounds facilitating skin regeneration [1]. 

Modern skin care methods and products, including moisturizing and lifting agents, can be divided into two groups. The first group includes cosmetic gels and creams containing oils, extracts, vitamins, and other biologically active components of natural origins (polysaccharides, proteins) and synthetic compounds. These products mainly influence the surface skin layer and do not eliminate the causes of aging; thus, their continuous (daily) application is required. The second group includes non-surgical methods of skin treatment and rejuvenation, e.g., laser, light, and radio-frequency methods.

Chitosan, a derivative of natural polysaccharide chitin, possesses a set of properties that facilitate its use in medicine and tissue engineering [3]. Due to several important features, such as biocompatibility, bioresorbability, and absence of cytotoxicity (which applies both to the polymer and the products of its biodegradation), chitosan-based materials can be used in the preparation of wound dressings [4], or treatment of deep skin injuries of various etiologies. The materials based on chitosan fibers demonstrate vapor and gas permeability, atraumaticity, and good mechanical and elastic properties; all these features provide efficient wound healing.

Approximately 40% of chitosan and its derivatives currently applied in cosmetology enter the composition of skin care products [5]. The chemical structure of chitin is close to that of mucopolysaccharides (heparin and hyaluronic acid). Chitosan, being a non-toxic and non-immunogenic compound, is widely used as a component of cosmetic preparations. 

Chitosan and chitin are included in skin care formulations owing to the following characteristics: antimicrobial and antioxidant activities, mucoadhesive, regenerative, and penetrating abilities. Casadidio et al. [6] explain the penetrating ability of chitin and chitosan by opening and destruction of epithelial dense contacts due to a decrease in trans-epithelial electric resistivity. The chemical aspect of this mechanism includes the electric interaction between the cell membrane and positive charges on chitosan molecules, which leads to the reassociation of the proteins attached to dense contacts. In an acidic medium, chitosan with protonated amino groups becomes a polycation, which subsequently can form ionic complexes with a wide range of natural or synthetic anionic compounds [6]. Thus, the presence of chitosan in skin care products facilitates more intense action of biologically active substances on the skin.

It is noteworthy that chitin and chitosan, as well as their derivatives, such as N-carboxybutyl chitosan and glycol chitosan, possess properties similar to those of hyaluronic acid [7,8]. Chitin, being an excellent moisturizer, supplies water and prevents skin dehydration. Water-retaining ability of pure chitosan depends on its molecular mass [9]. The introduction of alkyl chains into water-soluble chitosan opens new possibilities in cosmetology [10,11]. The moisturizing effect of N-succinyl chitosan [12] included in a cosmetic cream turned out to be better than that of hyaluronic acid [13]. Besides, this polymer is compatible with anionic and non-ionogenic surfactants present in cosmetics. N-carboxymethyl chitosan (a soluble carboxymethylated derivative of chitosan) was developed for the first time by Muzzarelli [14].

The presence of secondary hydroxyl groups in the chitosan structure enables one to introduce carboxymethyl groups into the molecule and creates active centers for the absorption and retention of moisture [15]. Chitosan derivatives containing carboxymethyl fragments are used in the cosmetic industry due to their film-forming, thickening, and emulsion-stabilizing properties [14,16,17]. Chaiwong et al. [11] studied the influence of the molecular mass of chitosan on its antioxidant and moisturizing properties; high solubility of chitosan in water (89%) and high viscosity of the solution was demonstrated. As for the prolonged moisturizing effect on human skin, carboxymethyl chitin and carboxymethyl chitosan are superior to hyaluronic acid (HA) as ingredients of cosmetic and clinical medicinal preparations [9,16]. Liping et al. [18] studied the complex of chitosan with vitamin C as a multifunctional primary product for use in cosmetics. This chitosan derivative possesses good antioxidant, moisturizing, antibacterial, and film-forming properties. The chitosan composition containing ascorbic acid and niacinamide enhances the proliferation of fibroblasts. The complex of chitosan with linoleic acid and retinyl palmitate facilitates the penetration of cosmetic substances into the skin. These chitosan-containing compositions are considered potential anti-aging skin care products [19,20]. Casadidio et al. [6] discuss the physico-chemical and biological properties of chitin and chitosan and their applications in cosmetics. Several research works were focused on the preparation of chitosan capsules for skin rejuvenation [21,22], bleaching [23], and remodeling. Morganti et al. [24] developed anti-aging cosmetic products using chitin nanofibril-hyaluronan nanoparticles as carriers for active ingredients. Shipovskaya et al. [25] obtained the chitosan-containing hydrogel on the basis of low molecular weight and high molecular weight chitosan samples and the mixture of acids (glycolic acid, lactic acid, aminocaproic acid). The produced chitosan-containing gel is applied onto the preliminarily cleaned face, neck, and decollete; a thin layer of the gel is uniformly spread on the skin surface, left for 5–10 min, then washed away with warm water, after which a moisturizing cream is applied. The procedure is performed once a day three times a week for 30 days. Examination of patients and analysis of the state of the skin by a dermatologist (immediately after the first application, 14 and 30 days after the beginning of treatment) showed a significant therapeutic effect resulting from the use of this chitosan-containing gel. The skin moisture increased by 1.6–2.6 times. The wrinkle depth decreased by 2.5 times on average in all patients. The skin color became pink, and its turgor and elasticity increased.

Morganti et al. [26,27] revealed that biodegradable nonwoven tissues based on chitosan were shown to be ideal and innovative carrier systems for biomedical and cosmetic applications. They may be used to make tissue scaffolds, which are able to restore the skin micro-environment, and improve both delivery and penetration of the active ingredients into the right skin layer [27]. The compositions of face-lifting serums contain biopolymers that fill in irregularities and wrinkles. Also, the composition may include substances capable of creating a modeling net on the skin surface, which tightens the contours of the face and neck.

Mucoadhesive and film-forming properties of chitosan suggested an idea of the development of a facial serum based on chitosan or composite fibers containing chitin nanofibrils. Chitosan becomes attached to skin cells and forms a very thin gas-permeable net film, which supports facial contours, smooths wrinkles, and improves moisture retaining properties of the skin.

The present work discusses the result of the research aimed at preparation of chitosan fibers and composite fibers filled with chitin nanofibrils, the preparation, and studies of the facial serum containing chitosan and composite fibers.

## 2. Materials and Methods

The base of the facial serum consisted of two components: (i) pure chitosan fibers or composite fibers containing chitin nanofibrils; (ii) the buffer solution containing lactic acid and sodium lactate (pH = 5.8). Chitosan and composite fibers were prepared using chitosan purchased from Biolog Heppe GmbH (Landsberg, Germany) with molecular mass Mm = (1.64 − 2.0) × 10^5^ and deacetylation degree DD = 92.4%. The aqueous dispersion of chitin nanofibrils (SRL Mavi Sud, Italy) (C = 20 mg/mL) was subjected to ultrasound treatment with the use of an IL10-0.63 dispergator (“Ultrasonic Technique–Inlab”, St. Petersburg, Russia) for 10 min [28]. Chitosan powder was added to the ultrasound-treated aqueous dispersion. The mixture was stirred for 30 min until swelling and partial dissolution of chitosan occurred; then concentrated acetic acid was introduced at continuous stirring, and its concentration was brought up to 2%. The chitosan concentration in solutions was equal to 4.0 wt.%, and the content of chitin nanofibrils was 0.5 wt.% with respect to chitosan [29]. This chitosan concentration (4.0 wt.%) provides the necessary viscosity of the spinning solution and is optimal for the coagulation spinning of fibers [30]. The composite solutions were stirred for 30 min, then filtered and deaerated for 24 h at a pressure of 10 kPa. The 4.0 wt.% solution of chitosan was prepared according to the above technique without the addition of chitin nanofibrils.

**Figure 1:**
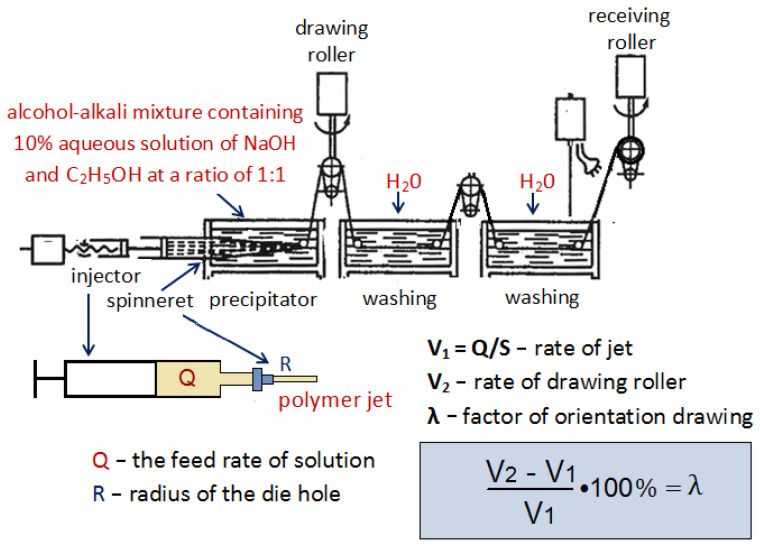
Scheme of chitosan fiber spinning.

The multifilaments were prepared by coagulation method using the laboratory equipment, which is schematically shown the Figure 1 [30]. The precipitant was the ethanol-alkali mixture containing a 10% aqueous solution of NaOH and C_2_H_5_OH (1:1) [30]. The die had 100 apertures 100 µm in diameter; the solution feed rate was 0.3 mL/min, the precipitation time was 150 s, and the factor of orientation drawing (λ) was 50%. The prepared filaments were rinsed with distilled water, dried in air at 50 °C, then stapled. The stapled fiber length was equal to 70 mm. The diameter of a single fiber was 10–15 µm.

Mechanical properties of the multifilaments (tensile strength, elongation at break, Young’s modulus) were investigated using an Instron 5943 setup for mechanical tests. The base length was 100 mm. Before testing, multifilaments were kept at normal climatic conditions (relative humidity 66%) for not less than 24 h.

Chitosan and composite fibers were studied by scanning electron microscopy on a SUPRA-55VP instrument (Carl Zeiss, Jena, Germany).

FTIR spectra were obtained with the help of a “Vertex70” spectrometer (“Bruker”) equipped with a ZnSe attenuated total reflectance (ATR) attachment (“Pike”); resolution: 4 cm^−1^, number of scans: 30. During registration of ATR spectra, the correction was made that takes into account penetration depth depending on wavelength.

The “Pike” attachment used in our experiments provides a uniform degree of clamping and, therefore, a uniform depth of penetration of IR radiation into the samples. Thus, the obtained spectra could be directly compared.

To study the properties of the basic serum, the stapled chitosan-based composite fibers filled with 0.5 wt.% of chitin nanofibrils (with respect to chitosan) were immersed into the buffer solutions consisting of the mixtures of lactic acid and sodium lactate (pH = 5.8). A drop of the viscous solution obtained 20–30 min after mixing of serum components was put onto glass support and left at room temperature until it dried completely, and a film was formed. The structure of the obtained composite films on supports was investigated by optical spectroscopy (Zeiss, Jena, Germany), by atomic force microscopy (a SOLVER PRO scanning probe microscope) in the contact mode. 

To estimate the cytotoxicity of the obtained composite fibers, they were incubated in a complete nutrient medium for 3 days. Before experiments, human dermal fibroblasts were seeded into the wells of a 96-well plate (5000 cells per well). Upon attachment of the cells onto the well surface (in 24 h), the medium was removed, and the nutrient medium obtained after fiber incubation was introduced into each well; the cell cultivation was performed for another 3 days. When the incubation period ended, the medium was removed, and the EMEM medium containing MTT (0.1 mg/mL) was introduced (50 µL per well). The cells were incubated in a CO2 incubator for 2 h at 37 °C. After removal of the supernatant, the formazan crystals formed by metabolically viable cells were dissolved in dimethylsulfoxide (50 µL per well), and optical density at 570 nm was measured using a plate spectrophotometer. The calculations involved polynomial regression analysis in Microsoft Excel.

## 3. Results and Discussion

To prepare a composite material based on a polymer scaffold filled with nanoparticles of various shapes, it is necessary to achieve uniform distribution of these particles in the scaffold. The degree of dispersion of nanoparticles depends on the dispersion method and the type of interaction between a polymer and a filler, which, in turn, is determined by the peculiar features of their chemical structures. In our previous works [28], the optimal method of ultrasound dispersion (v = 22 kHz, P = 630 watts, t = 10 min) and the best suitable concentration of chitin nanofibrils (0.3–0.5%) for preparation of composite fibers with good mechanical characteristics were selected. 

A study of the effect of an ultrasonic field on the process of dispersion of the chitin nanofibrils showed that the power and frequency of longitudinal vibrations in an aqueous medium determine the parameters of the cavitation process [28]. A model calculation showed that at a field power of 196 W, the radius of the air bubble changes smoothly, without destruction; the process is close to isothermal, which allowed us to conclude that the initial temperature changes in the cavity were weak. Increasing the power of the device to 630 W led to a sharp adiabatic compression and destruction of the air bubble, the temperature in the cavitation region could reach 507 °C leading to the destruction of the chitin macromolecule. These conclusions were confirmed by IR spectroscopy data: an increase in the duration of ultrasonic exposure up to 10 min caused a partial (approximately 13%) destruction of glycosidic rings with the formation of OH groups.

The results of light scattering, IR spectroscopy, and electron microscopy studies led to the conclusion that the optimal time for ultrasonic treatment of an aqueous dispersion of chitin nanofibrils was 4–10 min. For non-oriented film samples, the optimal chitin dispersion time was 4 min; in this case, chitin nanofibrils formed rather large flat structures about 200 nm wide, that were uniformly distributed inside the chitosan matrix. The deformation characteristics and strength of these specimens were improved. The strength of the composite films was 130 ± 11 MPa, their deformation was 43 ± 7.5%. After prolonged ultrasonic treatment (10 min), large particles are separated into individual nanofibrils. This processing time is optimal for obtaining composite monofilament with the best characteristics (strength 226 ± 4.8 MPa, tensile strain 10 ± 0.6%).

**Figure 2:**
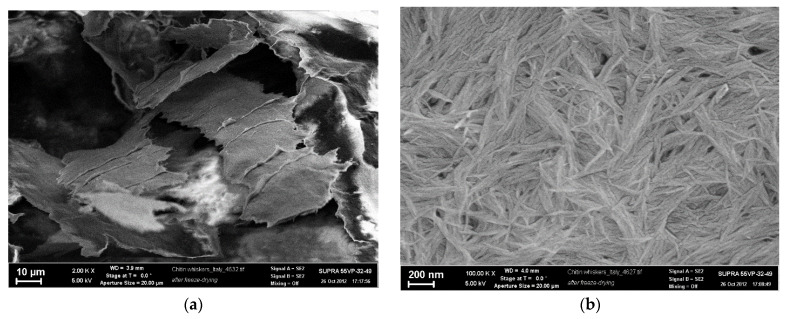

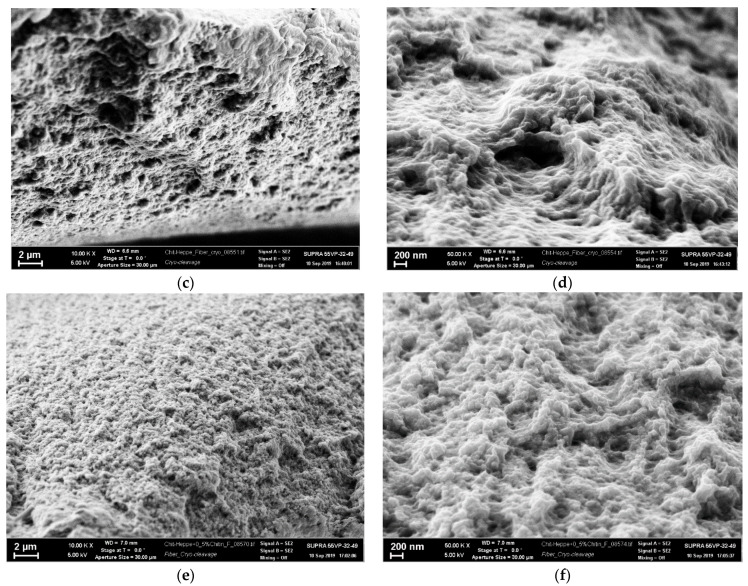
SEM images of the film obtained from aqueous dispersion of the chitin nanofibrils by lyophilization (**a**,**b**); cross section of chitosan (**c**,**d**) and composite filaments containing 0.5 wt.% chitin nanofibrils (**e**,**f**).

Figure 2 presents SEM microphotographs of chitin nanofibrils taken at different magnifications. The chitin particles were obtained by lyophilization of the initial aqueous suspension. As a result, a powder was obtained consisting of chitin microparticles with a width of about 30 μm and a thickness of 0.1 μm (Figure 2a), which, at higher magnification, turned out to consist of chitin nanofibrils with a width of ~20 nm and a length in the range from 600 to 800 nm (Figure 2b).

Figure 2 also shows microphotographs of the chitosan fiber (Figure 2c,d) and the composite fiber (Figure 2e,f) containing 0.5 wt.% chitin nanofibrils. As can be seen in Figure 2e,f, the composite fibers have a denser homogeneous structure compared to the unfilled chitosan fiber (Figure 2c,d), which is probably due to the good compatibility of the chitin nanofibrils and chitosan macromolecules due to the similarity of their chemical structure, that was explored in detail earlier elsewhere [28,31,32]. Chitin nanofibrils introduced into the chitosan solution facilitate the appearance of a new spatial arrangement of the chitosan chains. In the acidified aqueous solution, protonated chitosan chains preferably interact with functional groups located on the surface of rigid chitin nanofibrils and not with each other [28]. Our previous paper [33] presents a calculation performed by the molecular dynamics method. The calculation showed that the arrangement of chitosan macromolecules along an anisometric chitin nanoparticle is the most energetically favorable.

It was previously shown using molecular dynamics simulations that the energy of interaction between the chitosan macromolecules and the chitin nanofibrils facilitates good interaction between them [33]. Besides, it was demonstrated that the arrangement of the chitosan macromolecule in the longitudinal direction of the nanofibril is the most energetically favorable.

**Figure 3:**
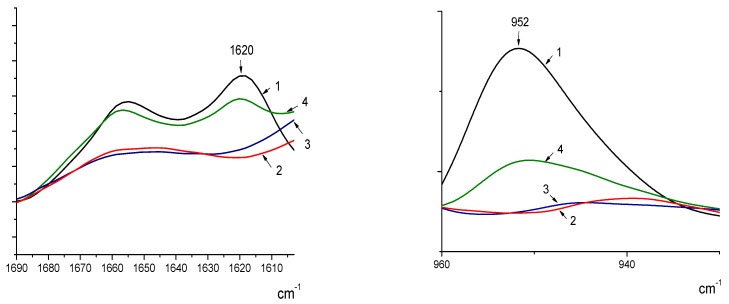
FTIR spectra of chitin (1), chitosan (2) and chitosan-based fibers containing of 0.5% (3), 30% (4) nanofibrils, respectively. The arrows mark the characteristic chitin bands: 1620 cm^−1^ (C=N vibration) and 952 cm^−1^ (valence vibrations of glucoside ring).

Figure 3 presents FTIR spectra of chitin (1), chitosan (2), and chitosan-based fibers containing 0.5% (3) and 30% (4) nanofibrils, respectively.

It can be concluded that the composite fibers contain both chitin and chitosan. This result becomes more clearly seen when the content of the chitin nanofibrils exceeds 10 wt.%. We have not observed any new bands that might be related to chemical interactions between particles and chitosan macromolecules. Thus, we have not revealed any chemical interactions between the chitin nanofibrils and chitosan macromolecules. The compatibility of chitosan and chitin nanofibrils is good because of the similarity of their chemical structure.

**Figure 4:**
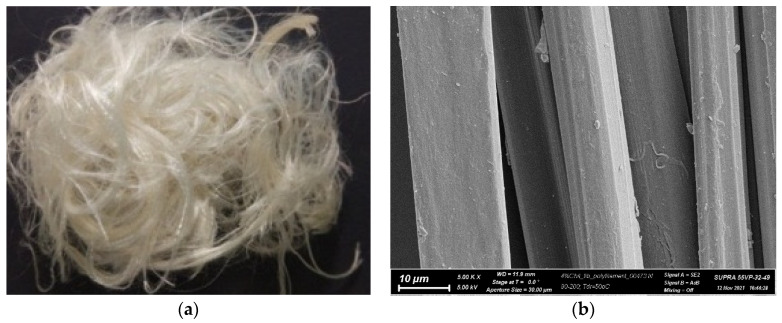
Digital image of stapled composite fibers (**a**) and SEM image of the multifilaments (filaments diameter: 10–15 µm) (**b**).

Figure 4 shows a digital image of the prepared fibers and their microphotograph obtained with a SUPRA-55VP scanning electron microscope (Carl Zeiss, Germany).

Orientation of the chitin nanofibrils and their interaction with the chitosan scaffold improve the strength and elastic characteristics of the composite chitosan/chitin nanofibril multifilaments (Table 1).

The dependencies of the mechanical properties of composite fibers on the content of chitin nanofibrils were discussed in [33]. These dependencies are nonmonotonic. In our previous works [29,33] it was shown that the maximum values of fiber strength and Young’s modulus are observed at a chitin concentration of about 0.3–0.5 wt.%. Indeed, the optimal filler content of 0.1–0.5 wt. % provides additional orientation of chitosan macromolecules on the surface of chitin nanofibrils on the one hand, and the sufficient mobility of chitosan chains for their orientation during drawing on the other. An increase in the concentration of chitin nanofibrils leads to the formation of a rigid network of particles (cluster structure), which, in turn, causes a decrease in the mobility of chitosan macromolecules [33]. In the composite fibers with this clustered structure, the processes of the orientation of chitosan macromolecules are rendered difficult.

**Figure 5:**
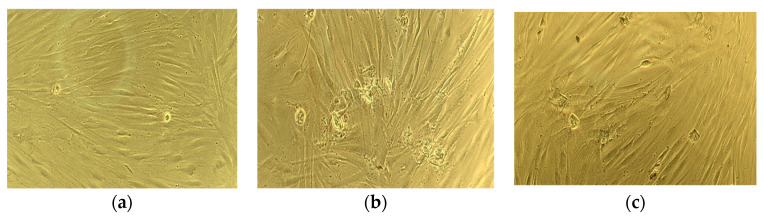
Microphotographs of human dermal fibroblasts after 3 days of cultivation: control sample (cultural plastic) (**a**); cells in the conditioning medium left after incubation of the chitosan-based fibers (**b**); cells in the conditioning medium left after incubation of the composite chitosan/chitin nanofibrils fibers (**c**).

**Figure 6:**
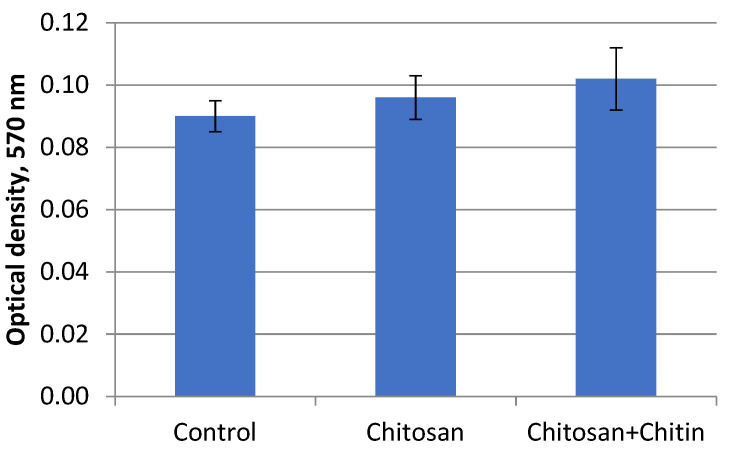
MTT test involving human dermal fibroblasts in the incubation medium.

Figure 5 and Figure 6 present the results of the biocompatibility studies of the chitosan and composite fibers containing chitin nanofibrils. 

The conditioned medium after incubation of chitosan-based fibers, as well as composite fibers based on chitosan and chitin nanofibrils, practically does not change the morphology of human skin fibroblasts compared to the control sample cells cultivated under standard conditions (Figure 5). After 3 days of cultivation, the cells formed a monolayer in all three variants. The cells have an elongated fusiform shape characteristic of fibroblasts. The data of the MTT test demonstrated the absence of a toxic effect of the conditioned medium on cell viability (Figure 6), which is confirmed by the data of other authors on the absence of a negative effect of chitosan on cell viability [34].

**Figure 7:**
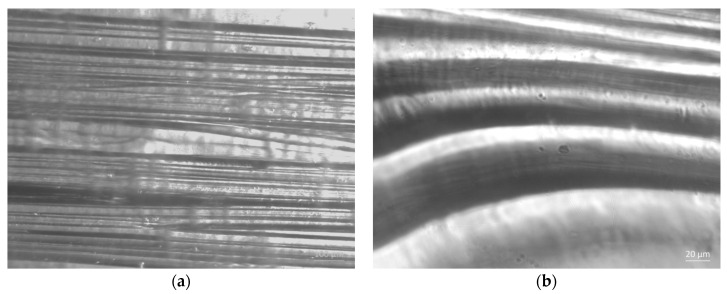
Optical photography of composite fibers based on chitosan and chitin nanofibrils at the time of application of a buffer solution pH = 5.8 (**a**); optical photography of fiber swelling for 15 min in a buffer solution (**b**).

Figure 5 shows optical images of the swelling of composite fibers in a buffer solution with pH = 5.8. The optical photographs (Figure 7b) show fiber-like oriented structural elements, which apparently are the fibers swollen in the buffer solution. This assumption is based on the fact that weak acids, including lactic acid, are good solvents for chitosan. Due to the presence of sodium lactate in the buffer solution the fibers do not dissolve completely. In addition, the integrity of fibers is maintained owing to the presence of chitin nanofibrils (which dissolve only in aprotic solvents) in the composite.

**Figure 8:**
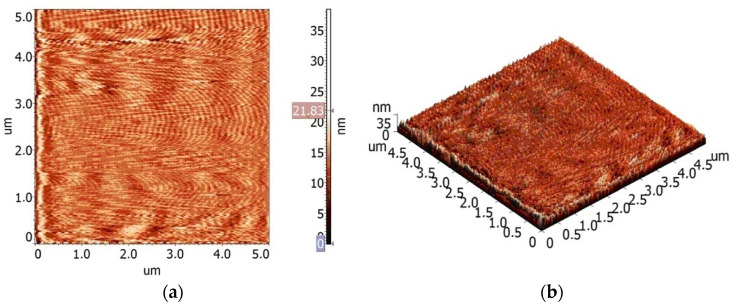

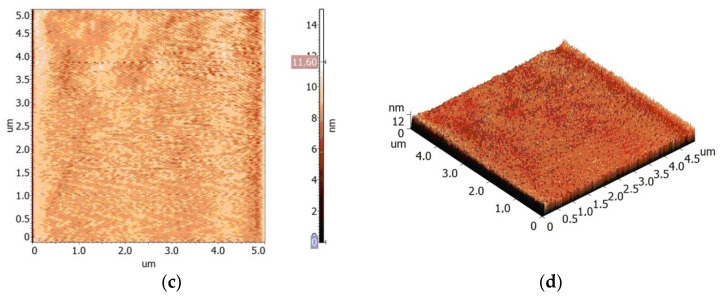
2D (**a**,**c**) and 3D (**b**,**d**) images of the surface topography of the films based on the buffer solutions containing chitosan/chitin composite fibers (**a**,**b**) and chitosan fibers (**c**,**d**).

To make a quantitative assessment of the relief of the films, obtained after drying on the glass of the composition “buffer solution (pH = 5.8)–composite fiber”, their free surfaces were studied by atomic force microscopy. Figure 8 presents 2D and 3D images the of surface topography of the films cast from the buffer solutions containing pure chitosan fibers and composite fibers.

**Figure 9:**
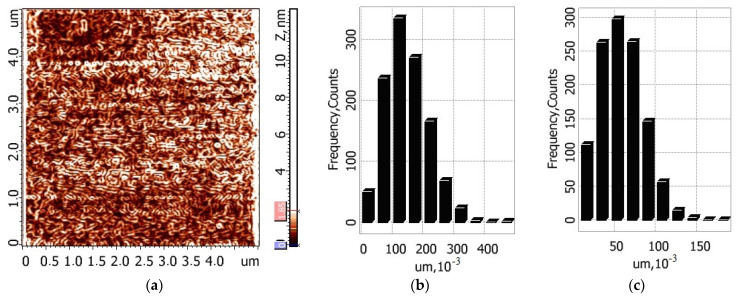
AFM image (the Gradient Image mode) of the surface of the film based on the buffer solution containing chitosan fibers (**a**) and histograms of length and width distributions of its structural elements (**b**,**c**).

**Figure 10:**
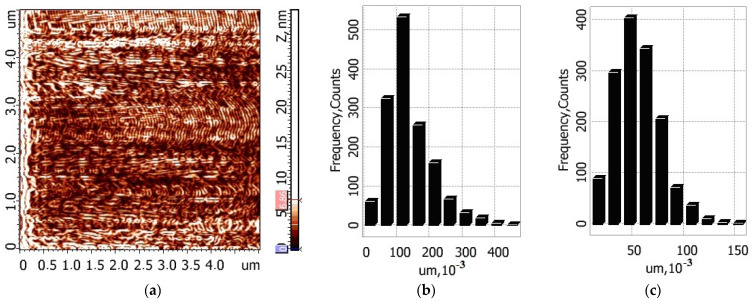
AFM image (the Gradient Image mode) of the surface of the film based on the buffer solution containing chitosan/chitin composite fibers (**a**) and histograms of length and width distributions of its structural elements (**b**,**c**).

The data processing software in the Grain section was used to process the AFM images of the surfaces of the studied samples in the Gradient Image mode, and histograms of length and width distributions for the structural elements of the studied surface were obtained (Figure 9 and Figure 10).

The oriented elongated structures can be seen in all images. According to the AFM data, the maximum ten-point height difference RZ for the surface of chitosan film is equal to 9.2 ± 2.2 nm; for the composite film, this value is equal to 27.9 ± 4.9 nm. This increase in surface roughness is possibly related to the presence of chitin nanofibrils in the composite fibers.

The obtained results indicate that the developed composite fibers possess film-forming properties. The peculiar feature of the obtained films is the presence of swollen oriented chitosan fibers, or “liquid filaments”, which serve as reinforcing elements and decrease film shrinkage in the process of drying the liquid composition at room temperature. This important property may have a considerable lifting effect upon the application of the serum to facial skin.

As shown by microscopic studies, the greatest relief of the film surface is observed after the introduction of composite fibers containing chitin nanofibrils into the buffer solution. Less swelling of such fibers in a mixture of lactic acid and sodium lactate compared to pure chitosan fibers enhances the reinforcing effect of the film as well as its lifting effect.

## 4. Conclusions

The composition with film-forming properties based on the buffer solutions consisting of lactic acid, sodium lactate, and stapled chitosan fibers or the composite chitosan-chitin nanofibrils fibers was developed. After drying at room temperature, the film contains swollen fibers (“liquid filaments”). Swelling of the composite fibers based on chitosan and chitin nanofibrils in this buffer solution is retarded, which causes the appearance of the reinforcing effect and the pronounced surface relief. In future work, we plan to conduct a series of preclinical studies to assess changes in skin properties after applying the serum containing composite fibers.

## Figures and Tables

**Table 1:** Mechanical properties of multifilaments.

Composition	Linear Density, tex	Young’s Modulus, GPa	Tensile Strength, MPa	Tensile Strain, %	Tenacity, cH/tex
Chitosan multifilaments	21.0	12.85 ± 0.9	160.59 ± 19.03	3.3 ± 1.33	10.95
Composite chitosan-chitin nanofibrils multifilaments	23.5	13.64 ± 1.62	196.97 ± 18.37	3.91 ± 0.81	13.62

## Data Availability

Not applicable.
